# Reducing Time Taken for Blood Test Requests on an Acute Inpatient Psychiatric Ward

**DOI:** 10.1192/bjo.2026.11379

**Published:** 2026-06-30

**Authors:** Madeleine Fletcher

**Affiliations:** Leeds and York Partnership NHS Foundation Trust, Leeds, United Kingdom

## Abstract

**Aims:**

To reduce the time resident doctors spend requesting blood tests on an acute psychiatric inpatient ward by introducing pre-printed patient identification (ID) stickers.

**Methods:**

Baseline timings for routine hand-written blood requests were recorded on an acute male inpatient psychiatric unit in Leeds. Using a fish bone analysis, root causes of inefficiency were identified. A PDSA cycle tested the intervention: ward administrators printed 2 pages of patient ID stickers for all new admissions which were then stored in the MDT room for use on blood request forms and bottles. A one-point lesson and step-by-step guide were distributed to all resident doctors and displayed in the doctor's office to support sustainability during staff rotation.

**Results:**

Mean time to request routine blood tests decreased from 7 minutes on average to 2.3 minutes after introducing patient stickers–an approximate 67% reduction–saving 3 hours 15 minutes per month across 28 routine requests on just 1 acute inpatient ward. Informal feedback indicated improved efficiency and a reduction in errors on request forms after introducing the ID labels. The process has been adopted locally and presented at the Adult Acute Inpatient Clinical Improvement Forum, where colleagues supported trust-wide roll-out.

**Conclusion:**

Pre-printed patient ID stickers reduced time and errors in blood requesting on an acute psychiatric ward. The intervention is low cost, sustainable and suitable for scaling across inpatient services.

